# Coral larval settlement induction using tissue-associated and exuded coralline algae metabolites and the identification of putative chemical cues

**DOI:** 10.1098/rspb.2023.1476

**Published:** 2023-10-18

**Authors:** Zachary A. Quinlan, Matthew-James Bennett, Milou G. I. Arts, Mark Levenstein, Daisy Flores, Haley M. Tholen, Lucas Tichy, Gabriel Juarez, Andreas F. Haas, Valérie F. Chamberland, Kelly R. W. Latijnhouwers, Mark J. A. Vermeij, Amy Wagoner Johnson, Kristen L. Marhaver, Linda Wegley Kelly

**Affiliations:** ^1^ Marine Biology Research Division, Scripps Institution of Oceanography, University of California San Diego, La Jolla, CA 92093, USA; ^2^ CARMABI Foundation, Piscaderabaai z/n, Willemstad, Curaçao; ^3^ Department of Marine Microbiology and Biogeochemistry, Royal Netherlands Institute of Sea Research (NIOZ), Den Burg, 1797 SZ, Texel, The Netherlands; ^4^ Department of Mechanical Science and Engineering, University of Illinois Urbana–Champaign, Urbana, IL 61801, USA; ^5^ Institute for Genomic Biology, University of Illinois Urbana–Champaign, Urbana, IL 61801, USA; ^6^ Department of Integrative Biology, University of Texas at Austin, Austin, TX 78712, USA; ^7^ Department of Microbiology, Radboud University, Nijmegen, 6525 XZ, The Netherlands; ^8^ Department of Biology, San Diego State University, San Diego, CA 92182, USA; ^9^ SECORE International, Hilliard, OH 43026, USA; ^10^ Department of Freshwater and Marine Ecology, Institute for Biodiversity and Ecosystem Dynamics, University of Amsterdam, Amsterdam, 1012 WP, The Netherlands; ^11^ Carle Illinois College of Medicine, University of Illinois Urbana–Champaign, Urbana, IL 61801, USA

**Keywords:** crustose coralline algae, coral larval settlement, settlement cues, metabolomics

## Abstract

Reef-building crustose coralline algae (CCA) are known to facilitate the settlement and metamorphosis of scleractinian coral larvae. In recent decades, CCA coverage has fallen globally and degrading environmental conditions continue to reduce coral survivorship, spurring new restoration interventions to rebuild coral reef health. In this study, naturally produced chemical compounds (metabolites) were collected from two pantropical CCA genera to isolate and classify those that induce coral settlement. In experiments using four ecologically important Caribbean coral species, we demonstrate the applicability of extracted, CCA-derived metabolites to improve larval settlement success in coral breeding and restoration efforts. Tissue-associated CCA metabolites induced settlement of one coral species, *Orbicella faveolata*, while metabolites exuded by CCA (exometabolites) induced settlement of three species: *Acropora palmata*, *Colpophyllia natans* and *Orbicella faveolata*. In a follow-up experiment, CCA exometabolites fractionated and preserved using two different extraction resins induced the same level of larval settlement as the unfractionated positive control exometabolites. The fractionated CCA exometabolite pools were characterized using liquid chromatography tandem mass spectrometry, yielding 145 distinct molecular subnetworks that were statistically defined as CCA-derived and could be classified into 10 broad chemical classes. Identifying these compounds can reveal their natural prevalence in coral reef habitats and facilitate the development of new applications to enhance larval settlement and the survival of coral juveniles.

## 1. Introduction

Coral larvae are known to actively select their settlement location [1–5] in response to biochemical cues [6,7], microbial biofilm composition and density [8,9], and abiotic chemical and physical cues such as salinity [10], light [11], turbulence [12] and external sounds [13,14]. Crustose coralline algae (CCA) are commonly described as beneficial members of reef ecosystems, producing chemical cues that coral larvae sense and use for both navigation [15] and metamorphosis [16,17]. CCA further provide advantageous conditions for juvenile corals to grow, inhibiting the growth of fleshy algae that smother and kill coral recruits [18]. However, when CCA are outcompeted by fleshy and turfing macroalgae, their positive impacts are diminished. Additionally, ocean acidification is predicted to reduce CCA growth [19–22]. To combat coral reef habitat degradation, active interventions that harness natural drivers of settlement could be employed in the future to promote coral larval settlement and coral recruit survival [3,4].

The role of CCA on the induction of coral larval settlement and metamorphosis has been widely studied [15,23–25] (further reviewed within [3,14]), with a large proportion of these studies targeting metabolites that putatively act as metamorphosis facilitators. Such molecules can be found within the extracellular matrix of CCA (tissue-associated), either produced by the organism itself or by its associated biofilm communities [6,7,16,26]. While tissue-associated CCA metabolites have been investigated in the past (e.g. [5]), metabolites that CCAs exude into the water column (exometabolites) are less well studied. One study simulated the effect of CCA exometabolites on coral larval settlement using a mixed assemblage of model monosaccharides in ratios representative of those found in a red algae, *Amansia* sp. [27]. However, the composition of the CCA exometabolite pool as a whole is incredibly complex and, thus far, not well studied [28]. To our knowledge, no study has ever tested the direct effect of CCA exudates (i.e. exometabolites) on coral larval settlement in the absence of microbes and the CCA organism.

The isolation of CCA-derived exometabolites allows for direct investigations of metabolite pool composition and larval settlement response as well as the potential to develop tools that enhance recruitment for active reef restoration [4]. To characterize the composition and role of CCA-derived metabolites in coral larval settlement and then extract these compounds into stable storage media for use in active restoration strategies, we conducted two experiments. First, we extracted tissue-associated and exometabolites from two species of CCA: *Hydrolithon boergesenii* and *Paragoniolithon solubile*. We supplied these metabolites to larvae of the elkhorn coral *Acropora palmata* [29], the mountainous star coral *Orbicella faveolata* [30] and the grooved brain coral *Diploria labyrinthiformis* [31] to evaluate the facilitatory role of CCA metabolites (both exometabolites and in the absence of the CCA organism and microbial communities). Second, we performed additional settlement assays with larvae of the boulder brain coral, *Colpophyllia natans* [32] to examine the efficacy of capturing CCA exometabolites for application within active restoration strategies. In this experiment, we used three distinct fractions of the CCA exometabolites to determine the optimal concentration and preservation method for inducing settlement. These experiments suggested CCA metabolites could be extracted and used in active reef restoration efforts.

## 2. Methods

### (a) Organism and gamete collection

**Figure 1.**
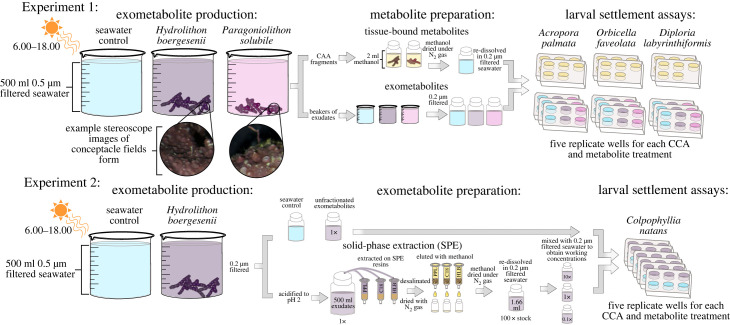
Experimental designs. Metabolites from CCA were harvested from the organism to examine larval response to CCA-derived compounds without the complication of other putative settlement mechanisms (e.g. bacterial lipopolysaccharides). Each experiment began with exometabolite production (left) by the organismal treatments: seawater control, the CCA *Hydrolithon boergesenii* and the CCA *Paragoniolthon solubile* (Experiment 1 only)*.* Stereoscope images (top left) of CCA tissue depict the conceptacle fields used to distinguish CCA species. Metabolite preparation (middle) in Experiment 1 (top) consisted of extracting tissue-associated metabolites from CCA with methanol which was dried and redissolved into filtered seawater. Additionally, the dissolved exometabolites produced by CCA were harvested and filtered for Experiment 1. In Experiment 2 (bottom), unfractionated exometabolites were harvested, filtered, then fractionated by SPE using three distinct resins. For the larval settlement assays, both tissue-associated metabolites and exometabolites were introduced to coral larvae in multi-well plates**.** Experiment 1 was run with larvae of the coral species *Acropora palmata*, *Orbicella faveolata* and *Diploria labyrinthiformis*. Experiment 2 was run with larvae of the coral species *Colpophyllia natans*.

Fragments of two species of CCA, *Hydrolithon boergesenii* and *Paragoniolithon solubile*, were collected at 10–15 m depth from the fringing reef in front of the Caribbean Research and Management of Biodiversity (CARMABI) Research Station, Willemstad, Curaçao (12°7′12″ N, 68°58′14″ W). Species of CCA were examined using a stereomicroscope and classified following protocols described in Ritson-Williams *et al.* [15]*.* Specimens of *H. boergesenii* were identified by the presence of low, dome-shaped conceptacles (figure 1), which occur across the CCA tissue in high abundance and density [2]*. H. boergesenii* is abundant across the Caribbean and has been frequently identified as a facilitator of coral larval settlement [24,33]. *P. solubile* was identified based on its smooth surface texture with large, steep conceptacles occuring at relatively low density (figure 1). This species has been shown to inhibit coral larval settlement; however, the exact mechanism of settlement reduction or inhibition is unknown [24]. During collection, we selected CCA specimens growing as rodoliths with the rubble almost entirely covered by the CCA organism. To ensure that only a single-algal species was present on each fragment, all collected fragments were trimmed using bone cutters to remove all other epiphytic macroalgae and encrusting organisms. To minimize potential stress on the CCA organism which could impact metabolite production, CCA tissue was not separated from the calcium carbonate rubble or the associated endolithic community. The fragmented CCA were allowed to recover from this tissue damage for 5 days prior to collecting the metabolites. During this recovery period, the CCA fragments were held in flow-through seawater aquaria at CARMABI Research Station, in an outdoor wetlab. Shading on the structure reduced irradiance to between 30 and 80 µmol photons m^−2^ s^−1^ [34]; these irradiance levels are similar to those in shaded coral reef alcoves at approximately 10–15 m depth, where these CCA are commonly found.

Gametes from four broadcast-spawning coral species were collected and larvae were reared following previously described methods [35–37], summarized below. In August 2019, *Acropora palmata*, *Orbicella faveolata* and *Diploria labyrinthiformis* gametes were collected 11, 6 and 11 days after the full moon, respectively. In September 2021, *Colpophyllia natans* gametes were collected 7 days after the full moon. *A. palmata* gametes were collected from the reef near the Curaçao Sea Aquarium (12°4′59″ N, 68°53′43″ W). *O. faveolata*, *D. labyrinthiformis* and *C. natans* gametes were collected at the Water Factory dive site in Curaçao (also known as Koredor; 12°6′34″ N, 68°57′23″ W). Gametes were mixed in 1 L polycarbonate fat separators within 1 h of collection in 0.5 µm filtered seawater (serial spun polypropylene sediment filters of pore size 50, 20, 5 and 0.5 µm; H2O Distributors, Marietta, Georgia). Following a 1 h incubation for fertilization and subsequent rinsing with filtered seawater, embryos were aliquoted into 1 l polystyrene clamshell food containers to achieve a density of less than or equal to 2 embryos ml^−1^. The next morning, all unfertilized eggs were selectively removed by pipette. To maintain larval health, larvae were transferred to clean containers and filtered seawater was replaced every 1–2 days until the start of settlement assays.

### (b) Production and collection of crustose coralline algae exudates

For both experiments, CCA specimens were allowed to release organic exudates over a single 12 h day period in acid-washed, glass beakers (figure 1). CCA incubations were conducted in the same outdoor wetlab where CCA were held during the recovery period. Each beaker was filled with 500 ml of 0.5 µm filtered seawater and locations were randomized within a single flow-through water bath, used to maintain stable ambient seawater temperature among treatments. All treatments (water control, *H. boergesenii* and *P. solubile*) were replicated (*n* = 5); the amount of CCA within each exudation beaker was approximated to cover the bottom of each beaker. Following the exudations, CCA surface areas within each beaker were approximated using calipers and general geometric shapes (e.g. cylinders, triangles, spheres, etc.): 141.98 ± 20.17 cm^2^ *H. boergesenii* and 124.48 ± 21.09 cm^2^ *P. solubile* in 2019 and 201.87 ± 89.59 cm^2^ *H. boergesenii* in 2021. Following a 12 h exudation period, CCA exudates were filtered through 0.2 µm polyethersulfone filter cartridges (Sterivex, Millipore, UK), after first flushing cartridges with 100 ml of sample. In 2019, filtered exometabolites were frozen at −80°C for use in coral larval settlement experiments or for extraction on Agilent PPL resin cartridges for liquid chromatography tandem mass spectrometry (LC-MS/MS) analysis. To extract tissue-associated metabolites, CCA fragments (trimmed to approximately 2 cm^2^ using bone cutters) were placed in triple acid-washed, and Milli-Q water rinsed 25 ml scintillation vials containing 8 ml of HPLC-grade methanol (figure 1). Five similarly treated scintillation vials contained only methanol to control for possible contamination of the vials. CCA fragments were removed from the methanol after 36 h, and the methanol was dried using nitrogen gas. Once the methanol was completely evaporated, tissue-associated metabolites were dissolved in 20 ml of 0.2 µm filtered seawater and stored in the dark for approximately 3 days, until settlement assays were started.

For Experiment 2 (September 2021), exometabolites and seawater controls were produced following the same approach as in 2019, but samples and controls were additionally acidified to pH 2 and extracted using three different, widely available, solid-phase extraction (SPE) resins (SPE resins: Agilent Bond Elut Priority PolLutant [PPL], Supelco HLB, Agilent Bond Elut C18; figure 1). C18 resins selectively bind non-polar compounds while PPL resins bind additional polar compounds from seawater [38]. PPL and HLB are both polymer-based sorbents which can have higher extraction efficiencies than monomer sorbents [39]. Additionally, HLB resins have been shown to extract lignin-phenols, oxygen-enriched and sulphureous compounds [39,40]. All three SPE resins were first cleaned and activated with HPLC-grade solvents following previously described methods [38]. After SPE, each resin column was dried with nitrogen gas and the resin-bound metabolites were eluted in HPLC-grade methanol. For the controls, clean but unused SPE resins were eluted in HPLC-grade methanol to control for possible interactions with background resin compounds or methanol carryover. Methanol fractions were dried under nitrogen gas in 20 ml triple acid-washed and Milli-Q rinsed scintillation vials and the resulting solids were then redissolved in 0.2 µm filtered seawater at a final stock concentration of 100×. This stock concentration was approximated under the assumption that 33% of the dissolved organic matter (DOM) would be captured on a PPL by redissolving the eluted metabolites in 1.66 ml filtered seawater [41]. After the addition of seawater, the rehydrated exometabolite fractions were stored in the dark for approximately 3 days to allow metabolites to fully resuspend in the water. Three additional PPLs from each treatment (CCA and seawater control) were frozen for analysis at the Royal Netherlands Institute for Sea Research (NIOZ).

### (c) Coral settlement assays

For both experiments, 10 ml of each exometabolite treatment (1× concentration) and 10 coral larvae were added to each well of a six-well polystyrene tissue culture plate with five replicates for each treatment. Plates were then haphazardly interspersed in a dark, temperature-controlled room (figure 1; 27°C). Larvae were introduced to the experiment once they were competent to settle, evidenced by their positive gravitaxis and interactions with the bottom surfaces of the rearing containers. This coincided with 4, 7, 3 and 3 days after the end of embryogenesis in *O. faveolata*, *A. palmata*, *D. labyrinthiformis* and *C. natans*, respectively. All replicate wells were examined daily for 3 days, and the number of settled larvae was recorded each day (figure 1). Successful settlement within both experiments was defined as larvae which were attached to the settlement wells but not necessarily fully metamorphosed.

Experiment 1: 13 days after the full moon (28 August 2019), larvae of *Acropora* *palmata*, *Orbicella* *faveolata* and *Diploria labyrinthiformis* were exposed to the two metabolites (tissue-associated and exometabolites) from both CCA species (*H. boergesenii* and *P. solubile*) and respective seawater controls for both the exometabolite incubation and tissue-associated metabolite extractions.

Experiment 2: 10 days after the full moon (30 September 2021), 10× stock exometabolites were pooled and mixed with 0.2 µm filtered seawater to create 0.1×, 1× and 10× treatments of each exometabolite fraction where 1× is the approximate concentration of these metabolites in the extraction beakers. Respective resin controls were prepared at 1× concentration. Experimental treatments then consisted of three exometabolite fractions (PPL, C18, HLB) at three metabolite concentrations (0.1×, 1×, 10×), unfractionated 0.2 µm filtered *H. boergesenii* exometabolites, unfractionated 0.2 µm filtered seawater controls and methanol extract controls of each SPE resin. Each treatment was tested to determine the effect on the settlement of *C. natans* larvae.

### (d) Liquid chromatography tandem mass spectrometry

Liquid chromatography tandem mass spectrometry (LC-MS/MS) analyses were conducted at the Royal Netherlands Institute for Sea Research (NIOZ) following previously described methods [38,42–44]. Extracts from PPL cartridges were eluted in LC-MS-grade methanol and injected into ultra-high-performance liquid chromatography (C18 core-shell column) electrospray ionization (ESI+) mass spectrometer (MS). A Q-Exactive orbitrap mass spectrometer (Thermo Fisher Scientific) in data-dependent acquisition MS/MS mode was used to collect fragmentation spectra within a range of 150–1500 mass-to-charge ratio (*m/z*) in positive ion mode at a MS1 resolution of 140 000 and MS2 resolution of 17 000 [43].

The informatic pipeline characterizes an ‘ion feature’ (also called molecular features) as an ion signal at specific retention times eluted off the UHPLC for which an MS/MS spectra is assigned. Ion features were detected and aligned using MzMine 3.2.8 [45] after raw LC-MS/MS files were converted to centroid-mzXML in MSConvert. Each ion feature is compared against all other ion features to identify structurally similar mass spectra using the Global Natural Products Social Molecular Networking (GNPS) platform [43,46,47]. GNPS clusters structurally similarly ion features together to form molecular subnetworks, which are collectively labelled a molecular network. Using the feature-based molecular networking framework within GNPS, we built subnetworks of structurally related features and matched spectra from our dataset to spectral libraries. We identified a single consensus annotation for each molecular subnetwork using the ConCISE platform and *in silico* predicted annotations from SIRIUS/CANOPUS [48–50]. All subnetworks were visualized using Cytoscape [51].

A total of 46 351 ion features were found from the multi-experiment LC-MS/MS run of 493 PPL extract samples collected from Curaçao. Experimental blanks were taken in parallel with sample PPLs and used to define background features using previously described methods [43,44]. Background ion features were defined as those for which the average sample log_10_-transformed extracted ion chromatogram (XIC) intensity was not greater than twice the max ion intensity measured in the blanks. If a molecular subnetwork had greater than 50% background ion features, the entire subnetwork would be flagged as a background feature network. Removing these background ion features and networks reduced the dataset to 33 634 features. Transient ion features were flagged as ion features that were present in only two or fewer samples with an XIC below 5E4; these were removed, which further reduced the dataset to 16 522 ion features. Subnetworks were defined as rare and removed if their max feature ion intensity was below the average ion intensity, further reducing the dataset to 16 000 ion features (3,160 subnetworks).

### (e) Statistical analyses

Settlement counts were relativized to total larvae added within each well and transformed with an angular transformation to approximate Gaussian distributions (electronic supplementary material, figure S1). All statistical tests and visualizations were designed in an internally developed R script (github.com*/z*quinlan/CCAExometabolites2023). A two-way analysis of variance (ANOVA) was used to evaluate the lone and interaction effects of CCA and metabolite source on per cent settlement within a coral species (asin(sqrt(percentage settlement) ∼ CCA species × metabolite source). A Dunnett's post hoc test was used to assess whether the tissue-associated or exometabolites of each CCA species induced significantly higher settlement rates than the seawater controls; this was tested separately for each metabolite type and coral species. For the fractionation experiment, two Dunnett's *post hoc* tests were run following the two-way ANOVA to test for significantly higher settlement rates relative to both the unfractionated seawater control as well as the respective column control.

The metabolomics data were analysed by subnetworks using log10-transformed XIC intensity. A random forest model with 10 000 permutations and 29 188 trees was used to compare subnetwork sum ion intensity between the seawater control and CCA treatments [52,53]. We characterized the major subnetworks driving treatment clustering within the random forest model as those subnetworks that had a mean decrease accuracy greater than or equal to one standard deviation plus the average mean decrease accuracy (electronic supplementary material, figure S1). To identify CCA-derived exometabolites, the subnetworks identified by the random forest model were then tested for significant differences between the seawater control and CCA treatments using a two-tailed *t*-test (log_10_(XIC)∼water). All reported *p*-values were corrected using the Benjamini–Hochberg false discovery rate (FDR) method [54].

## 3. Results

### (a) Larval settlement in response to unfractionated tissue-associated and exuded metabolites from crustose coralline algae

**Figure 2.**
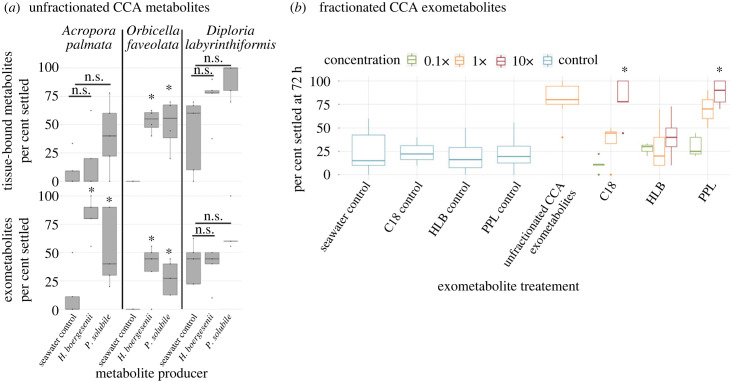
Larval settlement response to unfractionated and fractionated CCA metabolites. (*a*) Settlement of *Acropora palmata*, *Orbicella faveolata* and *Diploria labyrinthiformis* larvae introduced to unfractionated tissue-associated metabolites (top) and exometabolites (bottom) derived from the CCA species *Hydrolithon boergesenii* and *Paragoniolithon solubile*. Treatments that induced significantly higher settlement rates compared to the seawater control (Dunnett's FDR correct *p*-values < 0.05) are denoted by an asterisk. (*b*) Percentage settlement of *Colpophyllia natans* larvae on fractionated *H. boergesenii* exometabolites after 3 days. Concentrations of each exometabolite fraction were approximated with 1×, representing the initial concentration of these metabolites prior to fractionation using SPE resins (summarized further within the methods) and are coloured accordingly. Treatments that induced significantly higher settlement rates compared the seawater control and respective column methanol control are marked by an asterisk (Dunnett's FDR correct *p*-values < 0.05).

Larval settlement in response to CCA-derived metabolites was tested using three common Caribbean reef-building corals; *Orbicella faveolata*, *Acropora palmata* and *Diploria labyrinthiformis.* In the tissue-associated seawater controls, 0% of the *O. faveolata*, 8.48% ± 6.46% of *A. palmata*, and 41.3% ± 15.0% of *D. labyrinthiformis* exhibited a settlement response. In the exometabolite seawater controls, 0% of the *O. faveolata*, 12.2% ± 9.69% of *A. palmata* and 40.3% ± 7.93% of *D. labyrinthiformis* exhibited a settlement response (figure 2). *O. faveolata* was the only species to exhibit significantly higher larval settlement in response to tissue-associated metabolites than the seawater control (figure 2*a*; Dunnett's *p*-value < 0.0001). However, both *A. palmata* and *O. faveolata* larvae responded to exuded CCA metabolites with increased settlement (Dunnett's FDR *p*-value ≤ 0.0243). *D. labyrinthiformis* larvae did not exhibit significantly increased settlement rates compared to seawater controls, although more than 40% of larvae settled in both metabolite treatments (Dunnett's FDR *p*-value > 0.05).

### (b) Larval settlement response to fractionated *Hydrolithon boergesenii* exometabolites

To capture settlement-inducing metabolites in a form that could be more easily stored, analysed and potentially used in the future production of coral settlement substrates, we extracted exudates from *H. boergesenii* using three SPE resins: PPL, C18 and HLB. Larvae from *Colpophyllia natans* were exposed to these three exometabolite fractions, as well as unfractionated CCA exudates, and respective seawater controls for each resin type. After 72 h of incubation, the average settlement rate across all controls was 21.2% ± 18.41% (figure 2*b*). Unfractionated *H. boergesenii* exudates induced a larval settlement rate of 82.9% ± 5.77%. Exometabolite fractions from C18 and PPL used at 10× are the only fractions that induced significantly higher levels of settlement than both the unfractionated seawater control as well as their respective resin type controls (Dunnett's FDR *p*-value ≤ 0.045). Settlement rates induced by these two exometabolite fractions (C18 and PPL at 10× concentration) were comparable to the unfractionated CCA exometabolites, inducing larval settlement rates of 80.0% ± 10.18% and 70.0% ± 18.43%, respectively. All other treatments did not differ significantly from the control treatments (Dunnett's FDR *p*-value > 0.05).

### (c) Exometabolite composition of the crustose coralline algae *Hydrolithon boergesenii*

**Figure 3.**
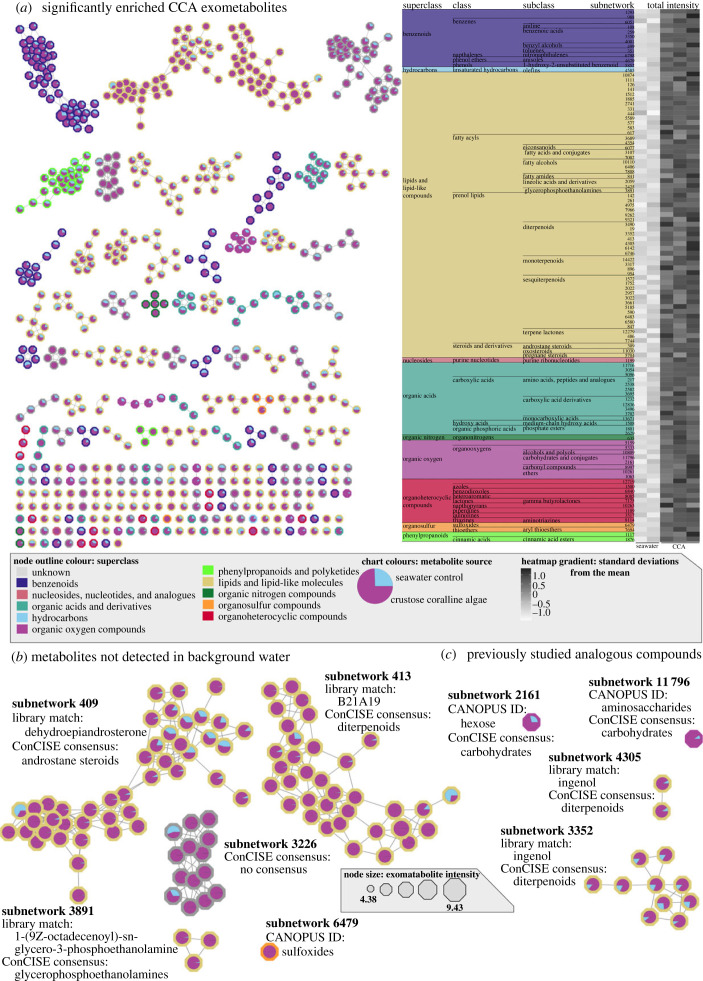
Chemidiversity of CCA exometabolites and putative chemical inducers of coral larval settlement. (*a*) Molecular network of significantly enriched metabolites (left) and the relative enrichment are depicted as a heatmap of standard deviations from the mean intensity (*z*-scored log_10_ XIC) and ordered by chemical ontology (right). Thirty-five subnetworks that could not be matched to library spectra nor identified by CANOPUS/ConCISE are not shown. (*b*) Subnetworks predominantly composed of molecular features that were absent within the seawater control at any intensity. (*c*) Four molecular subnetworks that are structurally similar to previously identified settlement and metamorphosis cues. Subnetworks are coloured by ConCISE consensus superclass with relative production intensity of each molecular feature for seawater control and CCA portrayed as a pie chart within each node. Node sizes of subnetworks in (*b*) and (*c*) are representative of metabolite sum intensity (i.e. XIC).

The exometabolites derived from *H. boergesenii* were characterized using untargeted liquid chromatography tandem mass spectrometry, spectral library matches and *in silico* structure prediction pipelines [35–38,43]. First, a random forest model was used to identify 696 subnetworks that were important for separating CCA exometabolites from the seawater control metabolite pools. Of the 372 subnetworks significantly enriched in either treatment (two-tailed t-test FDR corrected *p*-value < 0.05), 145 were enriched in the CCA treatment (figure 3*a*). These 145 subnetworks were classified into a wide variety of molecular families. Overall, 44.57% ± 0.34% of the ion intensity enriched in the CCA exometabolite pools were included in the lipids and lipid-like compounds superclass, 16.59% ± 0.09% were identified as benzenoids and 7.34% ± 0.08% were characterized as organic acids, with all other superclass categories comprising less than 5% of the CCA exometabolite pool. We identified five subnetworks that were only detected in CCA samples and that fell below our limit of detection in the seawater control samples (figure 3*b*; electronic supplementary material, figure S3). These subnetworks were classified as androstane steroids, diterpenoids, glycerophosphoethanolmines, sulfoxides and one unclassified subnetwork. Of all the metabolites enriched in CCA exometabolites, four of the subnetwork's annotations were analogues of previously identified compounds which played some role in swimming larvae settlement response (figure 3*c*). To provide a foundation for future research into exometabolite settlement cues and to facilitate the incorporation of settlement-inducing exometabolites into coral settlement substrates, a curated data frame of all significantly enriched *H. boergesenii* metabolites was organized into a table (electronic supplementary material, table S1). For each spectral feature, we present the production intensity (XIC), molecular weight (*m/z*), predicted elemental formula, predicted structure and any available library spectral match information (github.co*m/z*quinlan/ccaSettlement2023).

## 4. Discussion

Previous studies investigating the role of CCA in coral larval settlement have identified species-specific settlement behaviours in response to different CCA species and have characterized the microbial symbionts and biofilm-associated metabolites that may facilitate this process [7,15,24,25,27,28,55,56]. Currently, little is known about the compounds which are exuded into the water column by CCA and their role in facilitating coral larval settlement. Recent developments in methods for SPE of DOM from seawater and untargeted metabolomics [38,42–44] have enabled the characterization of this largely unknown and diverse pool of molecules. The aim of this study was to investigate the effect of CCA-derived tissue-associated metabolites and exometabolites on the induction of larval settlement in corals, characterize the composition of CCA exometabolites using untargeted metabolomics and to develop a method to capture and preserve CCA-derived exometabolites in order to make them available to coral breeding and restoration practitioners. While there were coral species-specific differences in response to the metabolites harvested from two species of CCA (*Paragoniolithon solubile* and *Hydrolithon boergesenii*), larvae from three different scleractinian corals—*Acropora palmata*, *Orbicella faveolata* and *Diploria labyrinthiformis*—were consistently induced to settle by the dissolved exometabolites rather than by tissue-associated metabolites (figure 2*a*). Further, it was shown that fractionated exometabolites from *H. boergesenii* could be isolated using SPE resins and still exhibit similar induction levels of larval settlement (figure 2*b*). Finally, liquid chromatography tandem mass spectrometry (LC/MS-MS) was used to characterize the exometabolites that were produced by *H. boergesenii* and captured using PPL SPE columns, thus identifying groups of molecular features (subnetworks) that potentially induce the larval settlement response (figure 3).

### (a) Tissue-associated and exuded metabolites produced by crustose coralline algae facilitate the settlement of coral larvae

Tissue-associated metabolites from both species of CCA enhanced the settlement success of *O. faveolata* larvae relative to the seawater control (0% versus 51.70%), but had no significant effect on *A. palmata* and *D. labyrinthiformis* larvae (figure 2*a*). By contrast, metabolites exuded by both CCA species improved the settlement success of both *A. palmata* and *O. faveolata* larvae by 5.5-fold and 1.4-fold, respectively. As tissue-associated metabolites and exuded exometabolites were isolated through different processes, the samples could contain variable carbon concentrations. Without bulk carbon measurements, comparisons between the two metabolite types are more nuanced. It is possible that the observed difference in settlement induction between exometabolites and tissue-associated metabolites could be the result of differences in concentrations. However, this caveat does not diminish the robust settlement induction from CCA exometabolites observed within this study as this direct settlement induction without microbes or live CCA has previously never been identified.

A positive response to metabolites extracted from the CCA species *H. boergesenii* was expected, given that previous studies have shown larval induction in response to this species [24]. However, we also observed a positive settlement induction by exometabolites derived from CCA species *P. solubile*, which was not expected. Prior studies have categorized the CCA species *P. solubile* as an inhibitor of coral larval settlement [24], and although the mechanism of inhibition remains undiscovered, others have posited that this response could be related to differences in microbial community composition, differences in metabolite production, or from tissue sloughing [24,28,57]*.* The findings presented here suggest that the metabolites from *P. solubile* facilitate settlement of coral larvae and the previously identified settlement inhibition is likely due to a different mechanistic pathway.

The CCA exometabolites showed a better induction of larval settlement compared to tissue-associated metabolites (figure 2*a*), though different coral species also demonstrated different requirements for settlement induction. For example, *D. labyrinthiformis* larvae settled in high proportions in the absence of CCA metabolites, suggesting that settlement in this species is less dependent upon chemical cue reception overall. Previous studies conducted in the Caribbean, northern Pacific and the Great Barrier Reef all demonstrated some CCA specificity for settlement induction [23,24,58]. These prior observations, combined with the variability in response to CCA metabolites in this study, suggest that perhaps chemical cues which induce larval settlement vary between coral species. It is also possible that a wide variety of compounds induces larval settlement rather than a single ubiquitous settlement metabolite. Coral species may also differ in their sensitivity to a given chemical cue. All these factors highlight the opportunity for further studies to examine the response of diverse coral species to organismal metabolites, whether they be derived from CCA or other reef constituents.

### (b) Fractionated crustose coralline algae exometabolites can induce similar levels of larval settlement

A global decline of coral reefs has evoked a need for effective restoration interventions [59–61]. One growing approach to coral restoration is the large-scale breeding and propagation of juvenile corals [59,60]. Typically, coral larvae are settled onto artificial tiles pre-conditioned in the ocean or in aquaria until colonized with settlement-inducing biofilms and CCA. However, this conditioning step can be time consuming, labour intensive, and can result in fouling by unwanted communities such as fleshy algae. Novel substrates with specialized topologies and additives that promote settlement and survival [62] are a promising avenue to improve the efficacy of this process. Settlement tiles such as these could be enhanced by incorporating natural settlement cues captured from the environment. The second experiment in this study illustrated the ability to isolate natural coral settlement cues on SPE resins (PPL and C18) and use these cues in isolation to induce settlement of coral larvae (figure 2*b*). This finding is particularly striking because SPE columns, such as PPL, generally only capture up to 30–40% of the DOM pool [38], reducing the total diversity of compounds supplied to the coral larvae when using this approach. Specifically, PPL and C18 columns are inadequate for capturing polar sugars, although they have a high affinity for non-polar compounds [41]. Polar sugars like monosaccharides are exuded in substantial amounts by marine algae, and these compound types are also known to facilitate the growth of copiotrophic microbial communities that are harmful to corals [28,56,63–65]. Thus, the selection against polar sugars in the metabolite isolation procedures described here could help to reduce the risk of enriching a harmful microbial community when using these metabolite pools to induce settlement.

### (c) Hydrolithon boergesenii exometabolite pool composition

The goal of the exometabolite characterization was to evaluate the chemidiversity within CCA exometabolites as there could be a suite of chemical cues associated with larval settlement that are exuded into the water, independent of the biofilm and tissue-associated compounds that play significant roles in metamorphosis. While we cannot yet distinguish a direct relationship between any individual metabolite and settlement induction, these data provide an overview of numerous putative settlement-inducing compounds, complementing and expanding on the known suite of compounds that have been previously studied in the context of coral settlement and metamorphosis induction [6,7,17,66].

Many of the previous studies in CCA-facilitated coral larval metamorphosis have focused on tetrabromopyrrole (TBP) [6,17,67,68]. It is known that TBP is produced by the gammaproteobacteria *Psuedoalteromonas luteoviolacea* and has been shown to induce coral larval metamorphosis, but not necessarily settlement of coral larvae, as Tebben *et al*. [7] reported induction of metamorphosis by TBP without settlement. The compound TBP was not observed within our exometabolite pools, which is not surprising given that *Psuedoalteromonas luteoviolacea* and TBP are both biofilm-associated [6]. Furthermore, our samples were run on the mass spectrometer in positive ion mode, which has a higher sensitivity for non-polar compounds and compounds with proton-donating functional groups, enabling broader characterization of compounds eluted from PPL resins. However, this methodology could be an alternative hypothesis to explain the absence of TBP within our dataset. Aside from TBP, other compounds such as lipopolysaccahrides (LPS) have been linked to the metamorphosis of the tubeworm *Hydroides elegans*, a common marine fouling organism [69]. Neither TBP nor LPS were identified in our CCA exometabolite extractions, however, 59 subnetworks were putatively classified as lipids and lipid-like compounds which could play a role in larval settlement (figure 3*a*).

Along with the wide range of lipids and lipid-like compounds, we identified five subnetworks only observed within CCA exometabolite pools. It is not known whether these five subnetworks play a direct role in coral larval settlement; however, compounds belonging to the same chemical classes of the subnetworks have been previously ascribed bioactivities in aquatic ecosystems. The single-node subnetwork 6479 was classified by CANOPUS as a sulfoxide; while this is too broad of a category to directly associate with identified bioactive compounds, sulphated compounds have been previously found to have large roles in settlement induction of *Agaricia humilis* larvae [70,71]. Subnetworks 409 and 413 were characterized at the Subclass level as androstane steroids and diterpenoids, respectively, both from spectral library matches. Both steroids and diterpenoids have previously been characterized from gorgonian chemidiversity studies as chemical defense mechanisms against both macroalgal overgrowth, and biofouling [72]. Specifically, briarane-type diterpenoids, 24-ketal steroids, and common steroids inhibited the settlement of barnacle (*Balanus amphitrite*) larvae [72]*.* To our knowledge, none of these highly specific CCA subnetworks have been previously shown to induce larval settlement, though we predict each group represents an interesting element in the chemical ecology of coral reefs and should be investigated further.

In addition to the subnetworks not detected in the water control, several metabolites belonged to compound classes which have been previously identified within larval settlement studies (figure 3*c*). In soft corals, some diterpenoids, specifically the phorbol ester, 12-*O*-tetradecanoylphorbol-13-acetate (TPA), induced larval settlement by activating the protein kinase C in the cytosolic signal transduction pathway [73]. Our CCA exometabolites were enriched in seven diterpenoid subnetworks. Although none were characterized as TPA by either spectral library matches or CANOPUS, both subnetworks 4305 and 3352 (figure 3*c*) were composed of features which were matched to ingenol, which belongs to the same chemical classification as TPA (level 5 ClassyFire chemical ontology: tigliane and ingenane diterpenoids). Outside of coral larval settlement, the chemical mediators of settlement and metamorphosis of other invertebrate swimming larval have been well studied [72,74–78]. Barnacle larvae metamorphosis was induced using extracellular polymeric substances composed of a series of mono- and polysaccharides including difucosyl-para-lacto-*N*-neohexaose, tetarose and trisaccharides [74]. Two molecular subnetworks in our dataset, 2161 and 11796, were characterized by CANOPUS as hexoses and aminosaccharides, respectively (figure 3*c*). Most compounds that have been characterized in the literature are linked with metamorphosis of swimming larvae (e.g. TBP). While there are compounds of the same chemical classification within the CCA exometabolites presented here, it is possible that the metabolites which induce settlement versus metamorphosis are distinct from each other. Though several of the compounds highlighted in this study have previously identified bioactivities within the context of coral larval settlement, additional experiments will be required to validate the direct relationships between specific compounds and the induction of coral larval settlement.

### (d) Future applications

Settlement and metamorphosis in marine ecosystems have been widely studied in numerous contexts outside of coral larval settlement [72,74–76]. Most of these studies have focused on surface-bound metabolites and those produced by microbial biofilms [6,7,17,55]. Recent developments enabling analysis of organic compounds through SPE and untargeted LC-MS/MS have expanded our ability to characterize chemical cues which influence the behaviour of swimming larvae that are not yet in contact with the settlement substrate [42–44,46,49,50,79]. The analysis presented here represents the first analysis of putative settlement cues exuded into marine waters. Although we envision these data supporting active restoration efforts in coral reefs ecosystems, they have additional utility in understanding the reproductive life histories of marine invertebrates, general larval behaviours and biofouling by expanding the known chemidiversity of settlement-inducing metabolites.

As active restoration efforts become more common in coral reef ecosystems, the ability to store stocks of settlement-inducing compounds has greater applicability. The SPE resins produced here enable long-term, stable storage of settlement-inducing bioactive metabolites while avoiding introducing some classes of compounds known to facilitate antagonistic microbial communities in larval cultures. These resin-captured metabolites remain stable during storage and can still enhance settlement once eluted. The ability to maintain metabolites in a more stable format for extended amounts of time provides a powerful approach to produce artificial substrates that enhance larval settlement [35,59,60]. To that end, future research identifying the specific compounds directly associated with settlement and metamorphosis would be beneficial so that these compounds can be produced synthetically rather than harvested from natural organisms.

## Data Availability

All data are available from github (https://github.com/Zquinlan/ccaSettlement2023). All mass spectrometry raw data are additionally available from Massive (https://massive.ucsd.edu/ProteoSAFe/dataset.jsp?task=94f594f10de246309a362121636a306f) and networked under the GNPS job (https://gnps.ucsd.edu/ProteoSAFe/status.jsp?task=955b2e3bd17c4f39b5f9bccfb43c00e1). Supplementary material is available online [80].
